# Evaluation of Fluorotype MTB for detection of *Mycobacterium tuberculosis* complex DNA in clinical specimens from a low-incidence country

**DOI:** 10.1186/1471-2334-14-59

**Published:** 2014-02-05

**Authors:** Sabine Hofmann-Thiel, Harald Hoffmann

**Affiliations:** 1 synlab MVZ Gauting, IML red, WHO Supranational Reference Laboratory of Tuberculosis, Robert-Koch-Allee 2, 82131 Gauting, Germany

**Keywords:** *Mycobacterium tuberculosis* complex, TB diagnostics, MTBC, NAAT, PCR, Nucleic acid amplification, Fluorotype MTB, Respiratory

## Abstract

**Background:**

With Fluorotype MTB (FT MTB, HAIN Lifesciences, Germany) a new semi-automated assay for detection of *M. tuberculosis* complex (MTBC) in clinical specimens has been introduced. In a prospective study, we evaluated the diagnostic performance of FT MTB in a routine diagnostic setting in a low-incidence country.

**Methods:**

A total of 1039 respiratory specimens received for routine mycobacteriology diagnostics were analysed by FT MTB. Results were compared to those of culture, microscopy and clinical diagnosis. 61 specimens were excluded from further analysis due to bacterial contamination of cultures.

**Results:**

FT MTB detected 52 of 59 TB specimens (45 culture-positive with MTBC, 7 with clinical diagnosis of TB). With 902 of 912 non-TB specimens (884 culture-negative, 18 with growth of non-tuberculous mycobacteria) FT MTB was negative; discrepant positive FT MTB results were found with 10 specimens. Overall sensitivity, specificity, positive and negative predictive values were 88.1%, 98.9%, 83.8% and 99.2%. Sensitivity rates for smear-positive and smear-negative TB specimens were 100% and 56.3%, respectively. Seven of 978 samples (0.7%) yielded invalid FT MTB results.

**Conclusions:**

FT MTB is a new accurate, half automated assay for rapidly diagnosing TB and suitable for larger series of samples. Performance characteristics were found to be similar to those of other commercial NAATs. Its sensitivity in paucibacillary, smear-negative specimens and its utility for TB diagnostics in high-incidence settings needs to be addressed in further studies.

## Background

Since the introduction of rapid, easy to use nucleic acid amplification tests (NAATs), tuberculosis (TB) diagnostics is shifting world wide towards molecular tests. Among commercial assays for the detection of *Mycobacterium tuberculosis* complex (MTBC), Xpert MTB/RIF (Cepheid, USA) is securing the pole position particularly in low- and middle income countries with high burden of multi-drug resistant TB (MDR-TB). Due to the fully integrated cartridge design, the simple and safe workflow, and the ability to simultaneously detect MTBC and rifampicin (RMP) resistance, Xpert MTB/RIF allows rapid TB diagnosis and drug resistance testing also at the peripheral health clinic level [1,2]. In fully industrialized countries however, these strengths do not prove similarly effective. First, TB incidence as well as RMP resistance rate is comparably low. In Germany, for example, TB incidence is still declining reaching 5.3% in 2011; MDR-TB rate is at around 2% [3]. Second, the price per shot is high compared to other commercial assays. Finally, TB NAATs are often processed in large batches in which Xpert MTB/RIF would not offer significant time savings. Therefore, semi-automated assays designed for larger series are more attractive for many mycobacteriology laboratories in low-prevalence settings. Widely used semi-automated assays are, for example, COBAS TaqMan MTB (CTM-MTB) (Roche, Germany), BD probeTEC ET (probeTEC, Becton-Dickenson, USA) or Gen-Probe Amplified MTB Direct Test (AMDT, Gene-Probe, USA). Most of them are able to detect MTBC with specificities of nearly 100% whereas sensitivities appear more variable, in particular with paucibacillar specimens [4-7]. In direct comparison studies, performance characteristics have been shown to be in the range of that of Xpert MTB/RIF [8-10]. While probeTEC and AMDT are demanding many open work steps with certain risk of cross-contamination, CTM-MTB is, so far, the only one considered to be semi-automated using a real-time PCR platform [5].

Most recently, a new semi-automated assay has been introduced for rapid detection of MTB complex from respiratory and non-respiratory clinical specimens. The FluoroType MTB (FT MTB, HAIN Lifescience, Germany) is a HyBeacon-based PCR assay performed on a Fluorocycler instrument allowing amplification and detection in a closed system. The aim of our present study was to comprehensively evaluate FT MTB for the routine detection of MTBC in respiratory specimens and to compare the results to conventional culture, microscopy and clinical data.

## Methods

### Clinical specimens

In total, 1039 respiratory specimens (390 sputa, 556 bronchial aspirates, 89 bronchoalveaolare lavages, 4 tracheal secretes) were included in the study. They originated from patients with suspected infection by MTBC or non-tuberculous mycobacteria (NTM) and were sent for routine mycobacteriology diagnostics between March and July 2012. The study protocol, involving the use of clinical specimens and human data, has been approved by the ethics committees of the Bayrische Landesärztekammer (no. 06043) and the Ludwig-Maximilian University of Munich (no. 437-12). In the scope of an open prospective study, consecutive specimens were taken provided that (i) sputum material was of sufficient quality, (ii) enough material was available to perform Fluorotype MTB assay in addition to requested routine analyses, (iii) material from the same patient has not been tested more than one time before and (iv) the material did not come from a known TB patient under therapy or with a known history of TB, provided this information was available at the time of enrolment.

All specimens were decontaminated using N-acetyl-L-cysteine (NALC)-NaOH. After concentration by centrifugation, the sediment was resuspended in 1.5 ml of 0.5 M phosphate buffer (pH 6.8) and inoculated in liquid (MGIT™, Becton-Dickinson, Heidelberg, Germany), Loewenstein-Jensen and Stonebrinck medium. Smears were prepared from this suspension, stained with Auramin O and visualized with a fluorescence microscope (magnification × 400). From the leftover suspension DNA was prepared and used directly for Fluorotype MTB assay or stored frozen.

Cultures were incubated for up to 8 weeks. In case a primary culture turned positive with acid fast bacilli, the isolate was identified by DNA line probe assays (Genotype CM, Genotype MTBC, Hain Lifesciences, Nehren, Germany) or by sequencing of the 16S rRNA gene.

### DNA preparation

Fully automated isolation of DNA was done using the GenoXtract automat (HAIN Lifesciences) and the GTX DNA/RNA Extraction Kit, according to the instructions of the manufacturer. In brief, 700 μl decontaminated material was transferred into a screw cap container and heat inactivated at 100°C for 20 min. Subsequently, pump-pipette-tip units, reagent cartridges, and elution containers were mounted in the GenoXtract machine. The protocol was started. After about 40 min the ready-to-use DNA was isolated in a volume of 100 μl elution buffer.

### Fluorotype MTB assay

Fluorotype MTB test is performed on a FluoroCycler (HAIN Lifesciences) and combines DNA amplification using specific primers and subsequent melting curve analysis in one instrument. First, PCR mixes were freshly prepared by combining 3 μl amplification mix A (AM-A) with 7 μl amplification mix B (AM-B) in the pre-amplification room I. Subsequently, 6 μl of isolated DNA was added in the pre-amplification room II. Controls included 6 μl of PCR-grade water (negative control) and 6 μl of control DNA C + FT MTB (positive control). PCR mixes were immediately loaded into the FluoroCycler. Results were available after 2 hours 10 min. The FluoroSoftware automatically analyzed melting curves for the amplification control (AC) (70.5°C +/- 3°C) and for MTBC (melting point 60.0°C +/- 3°C) and computed results as “no MTB complex DNA detected” (MTBC negative, AC positive), “MTB complex DNA detected” (MTBC positive, AC positive or negative), “not interpretable” in case of unspecific peaks, or “invalid” in case of failure of positive/negative controls or AC.

### Analysis of results

FT MTB results were initially compared to outcomes of the cultures. In cases of discrepant results, clinical, histological and anamnesis data of the patients were included in the final evaluation. For example, an initially discrepant positive FT MTB result (FT MTB positive, culture negative) was re-classified “true positive” if the patient was finally diagnosed as TB case by other means, i.e. by combinations of medical history, clinical examination, radiology, histopathology, and treatment success with anti-TB regimens. Invalid or not interpretable results were excluded from the final calculation of sensitivity, specificity and prospective values. Calculations of positive and negative predictive values were based on the detected prevalence of 6% (59/971), which is largely in agreement with prevalence values found in laboratories of many low-prevalence countries. 95% confidence intervals were determined using the Vassar-Stats calculator. Statistical analyses were performed using unpaired *t* tests on the OpenEpi version 2.3 platform. A *P* value of <0.05 was considered statistically significant.

## Results

### Comparison of FT MTB with culture data

In total, 1039 respiratory specimens were analysed by FT MTB and, in parallel, subjected to microscopy and culture. 908 of 1039 specimens (87.4%) were culture-negative, 18 (1.7%) yielded growth of NTMs (9 *M. intracellulare*, 4 *M. gordonae*, 2 *M. avium*, 2 *M. fortuitum,* 1 *M. abscessus*), and 52 (5.0%) growth of MTBC (51 MTB, 1 *M. caprae*) (Table 1). Of the latter ones, 42 specimens (86.7%) were smear-positive. Bacterial contamination of liquid or solid cultures was observed in 61 of 1039 (5.8%) cases. They were excluded from further analysis.

**Table 1:** Comparison of FT MTB and culture results

		**No. of specimens with FT MTB results**			
**Culture results**^**a**^		**Pos**	**Neg**	**Invalid**	**Total**
**MTBC**		45	7	0	52
Smear positive			42	0	0	42
Smear negative			3	7	0	10
**No MTBC**		17	902	7	926	
Culture negative		17	884	7	908	
Growth of NTM		0	18	0	18	
**Total**		62	909	7	978	

^a^MTBC, *Mycobacterium tuberculosis* complex; NTM, non-tuberculous mycobacteria; pos, positive; neg, negative.

Of the remaining 978 specimens, FT MTB results were negative in 909 (92.9%) and positive in 62 (6.3%) cases (Table 1). Seven (0.7%) specimens yielded invalid FT MTB results. 902 of 909 (99.2%) negative FT MTB results matched with culture: 884 specimens yielded negative culture and from 18 specimens NTM grew (Table 1). Discrepant results were found with seven FT MTB negative specimens, which were all smear-negative but MTB grew in liquid culture; they were considered false-negative. 45 of 62 (72.6%) FT MTB positive specimens were in agreement with culture results: they grew MTBC in culture and 93.3% of them (42/45) were smear-positive. 17 of 62 (27.4%) FT MTB positive specimens were culture-negative and thus considered “discrepantly positive”. All but one of them (94.1%) showed a smear negative microscopy.

### Analysis of discrepant results

All 17 discrepantly positive FT MTB derived from patients without positive culture from any materials sent to our laboratory, i.e. none of the patients were registered in our records as a TB case. Nonetheless, seven cases were diagnosed with TB after review of the patients’ charts. Consequently, we re-classified the results from 7 of 17 specimens as “true-positive”. Two of them were recovered from patients with clinically and histological confirmed tuberculosis although cultures consistently remained negative. Further two specimens were recovered from patients already receiving TB therapy at the time of specimen collection; one of them was smear-positive. The patients’ records indicated that other mycobacteriology laboratories reported positive cultures from previous specimens. Three specimens were obtained from patients with a recent history of TB. All three had already successfully completed TB therapy within the previous 10 months and requested now diagnostic work-up for the suspicion of relapse. The remaining 10 specimens with discrepantly positive FT MTB originated from patients without any documented history or diagnosis of TB and were finally classified false-positive (Table 2).

**Table 2:** Resolved results for sensitivity, specificity, PPV and NPV of FT MTB (culture and clinical diagnosis)

	**No. of specimens with indicated results by culture and clinical data (*n*** **= 971)**^**a**^							
	**TB (*n*** **= 59)**		**no TB (*n*** **= 912)**					
	**FT MTB pos**	**FT MTB neg**	**FT MTB pos**	**FT MTB neg**	**Sensitivity**	**Specificity**	**PPV**	**NPV**
**Smear microscopy**	**(%)**	**(%)**	**(%)**	**(%)**	**(95% CI)**	**(95% CI)**	**(95% CI)**	**(95% CI)**
Smear positive	43	0	0	3	100	100	100	100
	(82.7)	(0)	(0)	(0.3)	(89.7-100)	(30.9-100)	(89.7-100)	(30.9-100)
Smear negative	9	7	10	899	56.3	98.9	47.3	99.2
	(17.3)	(100)	(100)	(99.7)	(30.5-79.2)	(97.9-99.4)	(25.2-70.5)	(98.3-99.6)
**All**	**52**	**7**	**10**	**902**	88.1	98.9	83.8	99.2
					(76.4-94.6)	(97.9-99.4)	(72.8-91.5)	(98.3-99.6)

^a^TB, tuberculosis; pos, positive; neg, negative; PPV, positive predictive value; NPV, negative predictive value.

**Figure 1:**
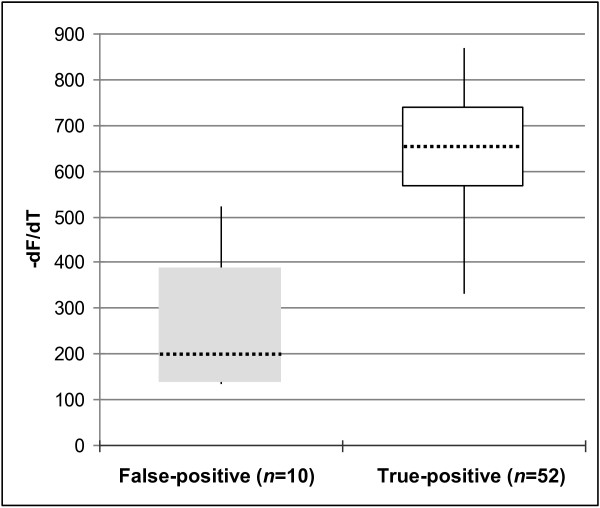
**Comparison of false-positive and true-positive results.** MTBC-specific melting curve peak values for false-positive (*left*, *n* = 10) and true-positive (*right*, *n* = 52) FT MTB results are shown. Boxes in grey (false-positive) and white (true-positive) represent means of values ± standard deviation, respectively. Maximum and minimum values are depicted by vertical lines. Dotted lines show median values.

Although FT MTB is a qualitative assay, signal values of melting curve peaks can be read from the corresponding graphs. Notably, false-positive results (*n* = 10) showed significantly lower MTBC-specific peak values (mean 257 units; standard deviation [SD] ±121.6 units) compared to that of true-positive results (mean 650 units; SD ±87.5) (*p* < 0.001) (Figure 1). All but one of false-positive results showed values below 320 suggesting the existence of a critical zone of “low grade positivity”.

### Sensitivity and specificity

After resolving discrepant and excluding invalid values, 10 out of 912 “no TB” specimens yielded positive FT MTB results corresponding to a specificity of 98.9% (Table 2). 52 out of 59 “TB” specimens yielded positive FT MTB results corresponding to an overall sensitivity of 88.1%. With smear-negative specimens, sensitivity was 56.3% (9/16), with smear positive ones 100% (43/43) (Table 2). Based on a prevalence of 6% TB specimens among the 971 study samples, positive and negative predictive values were calculated to 83.8% and 99.2%, respectively.

## Discussion

The FT MTB assay is a new semi-automated assay for detection of MTBC giving results within 3-4 hours. Similar to other semi-automated systems like COBAS TaqMan MTB, different workstations are required including DNA extraction, preparation of PCR mixes and amplification/detection. DNA extraction can be done automated using the GenoXtract instrument requiring only minimal hands-on time. Furthermore, amplification and detection is performed fully-automated in the Fluorocycler instrument; the handling of the instrument is simple and the analysis software designed user-friendly. Including all work steps, hands-on time for 12 specimens adds up to approximately 30 min being comparable to other semi-automated systems [10]. The Fluorocycler system is suitable for low numbers of samples as well as for large series, as up to 8 Fluorocycler instruments (each with 12 positions) can be operated simultaneously by one control computer. In comparison, the Xpert MTB/RIF system enables hands-on time of less than 3 min per specimens being of advantage only when handling a small number of samples.

The aim of the present study was to assess the performance characteristics of the FT MTB assay in a routine setting of a German mycobacteriology laboratory. FT MTB results from 978 respiratory specimens were compared to culture and clinical diagnosis as “gold standard”. The specificity (98.9%) of FT MTB was in the range of that of other NAATs (98.4%-99.7%, mean 99.1%) [2,4-6,11,12]. In particular, no cross-reactivity with 18 specimens growing NTM was observed. Ten positive FT MTB results remained unresolved, even after review of patients` clinical data. It remains elusive whether these false-positive outcomes are caused by residual MTBC DNA despite absence of active disease, as speculated before [13], or by unspecific amplification. Although reported as positive by the manufacturers’ analysis software, false-positive FT MTB tests seem to give lower MTBC-specific melting curve peaks than true-positive ones. Discrepant positive results in the “zone of low-positivity” are reported also for other NAAT systems [14-16]. A repeat testing is, therefore, generally recommended for specimens with low positivity values before transferring a final laboratory report to the clinical doctor.

The overall sensitivity of FT MTB reached 88.1% being in the upper range (63.2 – 95.0%, mean 84.2%) of levels reported for other commercial NAATs like BD ProbeTec ET (63.2-86.2%) [6,11], COBAS Taqman MTB (81.1-91.5%) [4,5], GeneProbe AMTD (95%) [12], Speed-oligo direct MTB (76%) [17] or Xpert MTB/RIF (88.0-92.2%) [2,18].

Considering smear-positive TB specimens only, sensitivity and PPV reached 100%. In comparison, sensitivity levels reported for other commercial assays were 95.5% to 100% [2,5,6,17-19]. A high positive predictive value for smear-positive specimens is particularly important in settings where NTM are common. Particularly in fully industrialized countries, NTM are increasingly isolated from clinical specimens and reported as cause of opportunistic infections mainly in immunocompromised individuals [20-22]. Therefore, modern NAAT assays should rapidly and reliably differentiate between TB and NTM infection, particularly in patients with immunodeficiency or in patients with first smear-positive sputa and unspecific clinical signs of TB. The FT MTB obviously fulfils this requirement.

Considering smear-negative TB specimens only, the sensitivity of FT MTB was 56.3%. Of the smear-negative TB samples which were missed by FT MTB, four exhibited positive culture results only after ≥ 24 days of incubation, indicating a paucibacillary nature of the specimens. This doesn’t mitigate the fact, that we found a sensitivity level for smear-negative samples which was in the lower range of values reported for other NAATs (49.1–86.7%, mean 64.1%) [2,4,6,12,17,18]. However, the low absolute number of smear-negative TB samples (*n* = 16) included in the present study might have biased the outcomes for such samples. Consequently, the sensitivity of FT MTB for paucibacillary specimens should be re-assessed in further, preferably multi-centre studies with higher numbers of smear-negative TB samples.

## Conclusions

In summary, FT MTB has proven to be a fast, easy and accurate new assay for direct detection of MTBC. The assay yielded good performance characteristic in terms of overall sensitivity and specificity with a potential weakness regarding sensitivity in smear-negative specimens which needs to be addressed in further investigations. Due to its user-friendly design, the closed system for amplification and detection and the possibility of simultaneously processing up to 96 samples without significant loss of time, FT MTB is an alternative to other semi- or fully automated assays particularly in low-prevalence countries.

## Competing interests

The authors declare that they have no competing interests.

## Authors’ contributions

SH-T supervised the study and laboratory work, performed data analysis and drafted the manuscript. H.H. participated in study design and participated in preparation of the manuscript. Both authors read and approved the final manuscript.

## Pre-publication history

The pre-publication history for this paper can be accessed here:

http://www.biomedcentral.com/1471-2334/14/59/prepub
